# Sources of knowledge—causation begets entropy increase, time progression, and information flow

**DOI:** 10.3389/fcogn.2026.1795815

**Published:** 2026-04-08

**Authors:** Georg Franz Weber

**Affiliations:** University of Cincinnati, Cincinnati, OH, United States

**Keywords:** causation, entropy, information, knowledge, time progression

## Abstract

The quest for knowledge has led to longstanding investigations into its sources. The foundation in information (data, evidence) is a cornerstone for the delineation of knowledge from belief or conviction. Through the execution of occurrences in the world, information flow is generated, which is observable and measurable in conceptual space with Lyapunov exponents and information dimensions. All occurrences have causes, which precede their effects, temporally and mechanistically. After much debate over the nature of cause-effect relationships, consensus has evolved, which interprets causation as the transfer of a preserved property (energy or momentum). This transfer modifies the thermodynamic entropy in closed and open systems alike. Independently, time progression has been characterized as being rooted in a transfer of heat (energy). Thus, causation is the driver of both, changes in thermodynamic entropy and time progression. It also effectuates a measurable evolution of information. With the acceptance of these interpretations, the underpinning of knowledge finds a unique definition. The essential causation of occurrences begets an information evolution, which—if observed in the process of scientific inquiry—serves as a basis for the generation of knowledge. Information flows not observed become lost to human access through dissipation in time and entropy.

## 1 Introduction

It is intrinsic in human nature that we seek knowledge about the facts (objects and occurrences) in the world. Whereas facts do not simply reveal themselves, access can be accomplished through the scientific process of inquiry, which acquires data (gathers information, accrues evidence) that are reflective of individual facts. In common usage, data are a collection of discrete or continuous values or properties that describe quantity, quality, or other characteristics attributable to facts. A datum is an individual value in a collection of data. Conventionally, measurements under the rules of logic and methodology produce such data (information). The more information is collected about facts through the application of scientific inquiry, the closer mankind gets to the truth (here understood as the entirety of objects and occurrences in the world). Thus, the nature of information and our access to it takes on central importance in epistemology (Weber, 2021).

Therefore, unique aspects in the acquisition and retention of information critically influence the nature of attainable knowledge. Any claim not based on information qualifies as conviction or belief, but not as knowledge. While knowledge does not equate to information, it is generated through the utilization of information at its base, and subjecting this base to interpretation (by forming mutually consistent/non-conflicting theories about the world) (Weber, 2013). Previous studies have linked the flow or evolution of information to dimensionality (of states, trajectories, or vectors) in conceptual space as well as to thermodynamic entropy (Weber, 2019, 2020). Separately, causation and time progression have been linked to thermodynamic entropy. These components suggest the hypothesis that *the energy transfer underlying causation leads to time progression and entropy changes; the information evolution associated with this process—if observed via scientific inquiry—can serve as a basis for the generation of knowledge. Yet, access to the evolving information is limited by dissipation in time and entropy*. Here, we analyze the propensity of these interconnected phenomena (causation, time progression, thermodynamic transform, information flow) to serve as a foundation for the generation of knowledge.

For the purpose of gaining knowledge, the interpretation of information is elaborated on the macroscopic scale. On this scope, accessible information can be generated and lost—one cannot unscramble an egg. This contrasts with the quantum mechanical principle of preservation of information (closely related to the concept of unitarity), which essentially means that the evolution of a closed quantum system is reversible and the underlying quantum information remains intact under transformation (Roncaglia, 2019; Chiribella and Scandolo, 2015). In the macroscopic world, information can dissipate (Gibbs, 1902; Goldstein, 2001; Zurek, 2003; te Vrugt, 2022; Rodríguez-Brea, 2025). As the information about the initial state of the system becomes more and more spread out, it is seemingly lost to the observer. The changes in accessible information are epistemologically relevant and quantifiable (Shaw, 1981).

## 2 Causes and effects

The nature of causation and the connection of facts via causality has for centuries been subject to substantial debate among thinkers of various backgrounds. Therefore, the scrutiny of causal connections among facts has been a foundational matter in the theory of knowledge. Causality denotes the influence by one fact (object or occurrence) on the outcome by another fact, where the cause is at least partly responsible for the effect, and the effect is at least partly dependent on the cause (the relationship is asymmetrical). X causes Y if, and only if, X precedes Y, and Y would not exist without X (antecedence and contiguity). Alternatively, an event E causally depends on C if, and only if, (1) C had occurred, then E would have occurred, and (2) C had not occurred, then E would not have occurred (Lewis, 1973). One cause can have multiple effects, and one effect can have multiple causes. The relationship of cause and effect constitutes a temporal as well as mechanistic connection. As only the future can be causally impacted, an effect of a prior fact can become a cause for an ensuing one, not a preceding one. Because causation requires the communication of a signal from cause to effect, and no signal can travel faster than the speed of light, any actual fact exerts causal efficacy that cannot propagate faster than light.

The Scholastic made a distinction between “propter hoc” (because) and “post hoc” (after). The empiricist David Hume disputed the justification for making reference to causal necessity, when all that is observable is regularity of occurrence between two events A and B. Immanual Kant sought to escape Hume's objections by postulating the “synthetic apriori.” As causality is not explicit in physical theories (e.g., for the equation F = m·a, it is interpretive to state that mass and acceleration are causative for generating a force of a certain magnitude or that a force is causative for imparting a certain acceleration on a given mass; the etiologic interpretation that gravity causes the constellations of planetary systems finds an alternative in the correlative interpretation that the constellations are explained by curvature of space-time). Hence, Comte and Mach suggested to consign related concepts to the realm of metaphysics. Later, Bavink (drawing on a classical, formal logic framework) sought to relegate causation to the realm of logical necessity and asymmetries in logical relationships (Bavink, 1954; Vollmer, 1985).

### 2.1 Energy transfer is the physical correlate to causation

As the 20^th^ century progressed, the evolutionary theory of knowledge accepted the proposition that causation is equivalent to the transfer of energy, thus offering a physical interpretation for causality (Lorenz, 1941; Vollmer, 1985, 1986). The effect receives energy from the cause in some form. Over time, certain refinements to this model have been discussed.

- For the frictionless, fully elastic collision between two balls of equal mass and velocity, it has been argued that the scalar kinetic energy $E_{kin}=\;\frac{1}{2}\;•mv^{2}$ is maintained, while only the momentum vector $\vec{p}=m\vec{v}$ is subject to a transfer (although an alternative interpretation sees an energy exchange of equal magnitude, which renders each collision member causative for the change of trajectory by the other). Hence, momentum transfer should be included in the definition of causation.
- More broadly, it has been proposed to expand the definition of causality to the transfer of conserved properties (“Erhaltungsgrößen”). David Fair argued for a physicalistic reduction of the causal relation to one of energy-momentum transference, suggesting that this approach can handle standard counterexamples to other analyses of causation, such as nomic and counterfactual dependencies. This perspective posits that causation involves the transfer of a conserved quantity between entities, thus providing a physical basis for understanding causal interactions (Fair, 1979).
- In quantum field theory, the term “microcausality” stipulates that only occurrences, which can be connected via a light signal, can exert effects on each other (Streater and Wightman, 2000).

A relationship of causality is confirmed by the documentation of an associated transfer of a conserved property (energy or momentum). Thus, causal necessity is more than logical necessity, it is empirically testable. Two occurrences, between which there is no energy transfer, are not directly causally connected. Viewed globally, all facts about the world are interconnected, and all energy in the universe is shared as well as constant (the law of energy preservation). The characterization of cause and effect is more refined. A direct causative link between A and B requires a transfer of energy from A to B. Notably, causation does not equate to determinism (Supplementary material 1).

### 2.2 Causation effectuates a change in entropy

Occurrences in the world impact thermodynamic and information entropy (Supplementary material 2). In equilibrative systems, entropy increases and accessible information is lost. In emerging systems, internal entropy decreases and attainable information is generated (Shaw, 1981). Frequently, causes are exerted by one system and the effects are received by another system.

- A transfer of energy (the definition of causation) is associated with a change in thermodynamic entropy, in open and closed systems alike. When heat (Q) is transferred to or from a system at a given temperature (T), the entropy of this system changes according to $\Delta S=\;\frac{Q}{T}$. This is so, because heat transfer affects the energy distribution among the particles in the system, altering its level of disorder.
- In idealized, reversible processes, work can be done without generating entropy. However, in real-world scenarios, irreversibilities (such as friction) always lead to some entropy generation.
- With a cause exerting its effect, prior uncertainty over its outcome is removed, and information is generated, which is measurable according to Lyapunov exponents and information dimensions. Causation is—at the very least—sufficient for generating information, the observation of which can produce knowledge.

*Causes lead to effects via the transfer of a conserved property (energy or momentum). Associated with the execution of every cause exerting an effect in the world is a quantitative change in entropy. An information flow takes place that is amenable to observation and measurement*.

## 3 Time progression

Time is the continued sequence of occurrences that present themselves in a succession from the past, through the present, into the future (Supplementary material 3). With the recognition that time is not a fundamental physical phenomenon (physics describes most processes as reversible; irreversibility was introduced by thermodynamics), a debate has ensued over the proper characterization and the underlying mechanisms of time progression. It has been recognized that processes experience their own internal time advancement, while our clocks measure averages over those.

### 3.1 Time progression is linked to the causation of occurrences

The direction of time, or the distinction between past and future, is fundamentally connected to thermodynamics [“the physics of becoming” (Prigogine, 1980)], and whenever a difference is manifested between past and future, a transfer of energy is involved. Only where there is heat is there a difference between past and future (“the past behaves like the future in absence of heat” Rovelli, 2018). The understanding, that time progression—internal to a system—is based on a transfer of energy, has linked it to causation in several ways:

- Every cause that exerts an effect also prompts (through the transfer of energy or momentum) an increment of time advancement.
- The signal from cause to effect cannot travel faster than the speed of light (299,792,458 meters per second), thus requiring a time interval for completion.
- Photons are carriers of time. Energy and period/frequency are properties of the photon [Equations 1, 2]

$$\begin{array}{l} E=h•f \end{array}$$ (1)

E = photon energy, h = Planck's constant, f = frequency of oscillation. In the rearranged form

$$\begin{array}{l} h=E•t \end{array}$$ (2)

time (t) is linked to energy. The period of a quantum may be interpreted as the unit of time. As the photon wavelet propagates, time and energy move at the speed of light. The quantum of action carries both (energy and time), a change in energy (causation) relates to the progression of time (Annila, 2021).

### 3.2 There exists a connection between time and entropy

If we were able to consider all properties, every possible initial state of a system would be a unique state. If every particle could be considered individually, all initial conformations of the system would be equivalent. The definition of entropy requires a coarse-graining in the description of a physical system (i.e., the distinction between micro-states and macro-states). The entropy of a macro-state is determined by the number of corresponding micro-states. Heat and entropy are assessments of a statistical depiction of nature. The distinction between past and future is intimately linked to this coarse-grained explanation (the difference would vanish in a detailed description of the microscopic state of the world).

Entropy (Supplementary material 2) has acquired a fundamental importance in physics. Entropy production, dissipation, and statistical irreversibility are deeply connected to the arrow of time (Spinney and Prokopenko, 2016). The continual development of a closed thermodynamic system, at any given temperature, from lesser to greater entropy (in equilibrium thermodynamics the asymptotic approach toward the state of maximal entropy) defines an arrow of time, because heat never passes from a cold body to a hot one (Rovelli, 2018).

A physical system progresses to the state of highest entropy. Although reversion is not impossible, Boltzmann showed that it is so unlikely that it may not arise throughout the lifespan of the universe (Boltzmann, 1964). Within the framework of conventional physical theories, entropy—strictly speaking—is a time-independent state function, which describes a system progressing to its most probable state. This is so, because time is not contained as a parameter in any of the formulas for entropy (Ben-Naim, 2020). However, once the concept is accepted that a transfer of energy equates to the progression of an internal time by the system, then entropy itself is the term for progression of the internal time associated with an occurrence. For closed systems, this internal time parameter is given by the second law of thermodynamics with the Δ in ΔS ≥ 0. Other attempts have been made to link canonical time to entropy (Supplementary material 4), they are not necessitated.

*Time progression is associated with a transfer of energy. This underpinning mechanistically links time to cause-effect sequences. It also associates time progression with entropy changes. Entropy itself is interpretable as an internal time parameter*.

## 4 Information

As indicated in the preceding sections, causes effectuate occurrences, the execution of which is associated with an evolution of information. The information content of an occurrence is explainable as being inversely related to its probability of manifestation; less probable occurrences carry more information, while more probable occurrences carry less information. With the event's execution, the previously unknown elements are removed and new information is communicated. An occurrence with a certain outcome yields no new information. For the acquisition of knowledge, measurements of these information changes are important.

Early algorithms for quantifying the information content of a manifestation were developed by Claude Shannon (Shannon, 1948). The basic model describes communication between a sender and a receiver via a channel, the capacity of which is calculated from its noise characteristics and constitutes an essential determinant. Random processes have an irreducible complexity, below which the signal cannot be compressed. The ultimate data compression is called entropy. Information is affected by its physical representation and communication is directed by thermodynamic driving forces via mechanisms of energy transduction (Karnani and Annila, 2009). More recently developed algorithms (Lyapunov exponents and information dimensions) may be better tailored to the study of physical systems.

### 4.1 Occurrences produce a dynamic of accessible information

Accessible information, once produced, is not stable for the duration. In both, equilibrative as well as emergent systems, such information has a limited lifespan.

- The second law of thermodynamics states that a closed system progresses toward a state of maximal entropy (the most probable state). Once this state is reached, all information on the path that led to it has become unretrievable, and uncertainty about its future is minimized. A rolling ball must eventually come to a stop. Once at rest, the history of its movement cannot be reconstructed by any means. Equilibrium thermodynamics has established the inescapability of losing attainable information about the past.
- The energy of physical systems is describable on the macroscale, which in classical mechanics is completely intelligible and can be captured with deterministic algorithms, vs. the micro-scale of thermal motion, which is randomly distributed and can be expressed only as averages over large numbers. Before the ascent of non-linear systems research, the states of the microscales were assumed to be stochastic and to constitute a lower limit of feasible explanation (McKelvey, 1998). An adjustment of this model was required in non-linear events that arise far from equilibrium, where energy is believed to emerge from the microscales and affect the macroscopic outcome (Shaw, 1981). In turbulent flow, information is continuously generated through an exchange between the micro- and macro-scales (it contrasts with laminar flow, where motion is governed by boundary and initial conditions, and no new information is generated, as outlined above). In such emergent systems, new information is constantly produced to replace all old information. The information generated anew by turbulent systems precludes prediction past a certain time, when new information has accumulated to entirely displace the initial data. Hence, in non-equilibrium thermodynamics, access to old information is lost.

The obliteration of accessible information about the history of a system on the macroscopic scale is a physical inevitability, whether the occurring process evolves toward equilibrium or toward emergence. In equilibrium systems, observable information is constantly lost. In complex systems, new information is generated to replace all old information (Shaw, 1981).

Any occurrence in the world can be characterized by its occupancy of a conceptual (abstract, hypothetical) space, the number of coordinates (degrees of freedom) of which is determined by the complexity of that manifestation. A comprehensive analysis in conceptual space (state space, phase space, or event space) releases the restriction of dimensions from to the axes of concrete space.

In physics, occurrences are frequently depicted as a flow along trajectories in phase space (in dynamics, motion often maps momentum vs. position variables, sometimes velocity vs. position) (Prigogine, 1980; Thompson, 1986). Uncertainty and information have been studied for such trajectories. Information flow can be quantitatively analyzed through the application of the multiplicative ergodic theorem, also called the Lyapunov characteristic exponent (Shaw, 1981). The average rate of information production associated with each step in the execution of the occurrence [bits of information per unit time, denoted as $\bar{\lambda }$ (Oseledets, 1968)] is a readout for the rate of divergence between trajectories. $\bar{\lambda }\;$is a system state function that remains invariant under coordinate transformations (Shaw, 1981). Stable periodic orbits have a $\bar{\lambda }\;$that is negative. The transition of a system from laminar to turbulent implementation is reflected in a conversion of $\bar{\lambda }\;$from negative (the event is an information sink) to positive (the event is an information source). The application of $\bar{\lambda }\;$to phase space trajectories yields a quantitative assessment of the information flow (for the information evolution of vectors in event space, see Supplementary material 5) associated with each step in the execution of the event.

### 4.2 Information evolution, measured in conceptual space, is tied to entropy

From abstract space descriptions, it is possible to calculate a fractal dimensionality, which can become reflective of certain characteristics that are attributable to the occurrence, such as its complexity, its entropy, and its evolution of information. On this conceptual basis, various readouts assess the information content (Shannon entropy, Renyi entropy), the evolution of information by a process (Kolmogorov-Sinai entropy), or the entropy rate (Kolmogorov-Chaitin complexity). Several of them (such as the Renyi dimension for information content or the Lyapunov dimension for information evolution), are interpretable as entropy, as information, and as dimension in abstract space (Weber, 2020). They lay the groundwork for computations of the information evolution by the occurrence in its entirety.

Mandelbrot (Mandelbrot, 1983) defined fractal dimension (occupancy of an assigned space) as a ratio that provides a statistical index of the complexity attributable to a state, a process, or an event. Derived therefrom, the assessment of complexity (assignment of a fractal dimension) [Equation 3] by an occurrence in state space, phase space or event space enables a direct connection to the associated information flow through the information dimension,


$$D_{\text{I}}=\underset{\varepsilon \to 0}{\lim}\frac{-\langle log\;p_{\varepsilon }\rangle }{\log\frac{1}{\varepsilon }}$$ (3)

D_I_ considers how the average information—needed to identify an occupied box—scales with box size, ε stands for the edge length of the box (the scaling factor), p is a probability. Closely related is the Rényi dimension of order α that is defined as [Equation 4]


$$D_{\alpha }=\underset{\varepsilon \to 0}{\lim}\frac{\frac{1}{\alpha -1}\log\left(\sum_{i}p_{i}^{\alpha }\right)}{\log\varepsilon }$$ (4)

where the numerator is the Rényi entropy of order α. The Rényi dimension is a generalization of the Shannon entropy. Thus, the Rényi definition connects uncertainty to the dimensionality of the space in which it is measured.

The information generated or obliterated by a given iterated map is a topological property associated with the connectivity of that map, which is determined by the underlying physical process. In a phase space that describes y as a function of x [y = F(x)], the average information change $\bar{\lambda }\;$over an interval is an integral weighted by the probability density P(x). When including the time per iteration, T(x), this formula becomes [Equations 5, 6]


$${\text{λ}}_{t}=\underset{n\to 0}{\lim}\frac{1}{n}\sum_{1}^{n}\frac{log_{2}|\frac{dy}{dx}|}{T_{n}}$$ (5)

where T_n_ is the length of time taken by the n^th^ iterate (Shaw, 1981). For occurrences, a dimensionality of the occupied conceptual space can be calculated, the magnitude of which equates to a function describing the generation of information. The Lyapunov dimension D_KY_ represents an upper bound for the information dimension of the system.


$$\begin{array}{l} D_{KY}=\;k+\;\overset{k}{\underset{i=1}{\sum }}\frac{{\bar{\lambda }}_{i}}{\left|{\bar{\lambda }}_{k+1}\right|} \end{array}$$ (6)

k = maximum integer such that the sum of the k largest exponents is non-negative, $\bar{\lambda }\;=$ Lyapunov characteristic exponent. This dimensionality gives an estimate of the Kolmogorov-Sinai entropy, which defines the information change inherent in the execution of the process. Pesin demonstrated that when the Kolmogorov-Sinai entropy is greater than 0, the dynamical system will display non-linearity. In the framework of Pesin's theorem, the sum of all the positive Lyapunov exponents gives an estimate of the Kolmogorov-Sinai entropy.

*For the attainment of knowledge, it is critical to collect information about occurrences via scientific observation. The information evolution (generation or obliteration) of an occurrence can be described at each step through the Lyapunov characteristic exponent and overall through fractal dimensions in conceptual space. These readouts enable quantitative assessments, thus measuring the basis for attainable knowledge. Applicable formulas allow representations of entropy as fractal dimensions in conceptual space*.

## 5 Implications for epistemology

**Figure 1:**
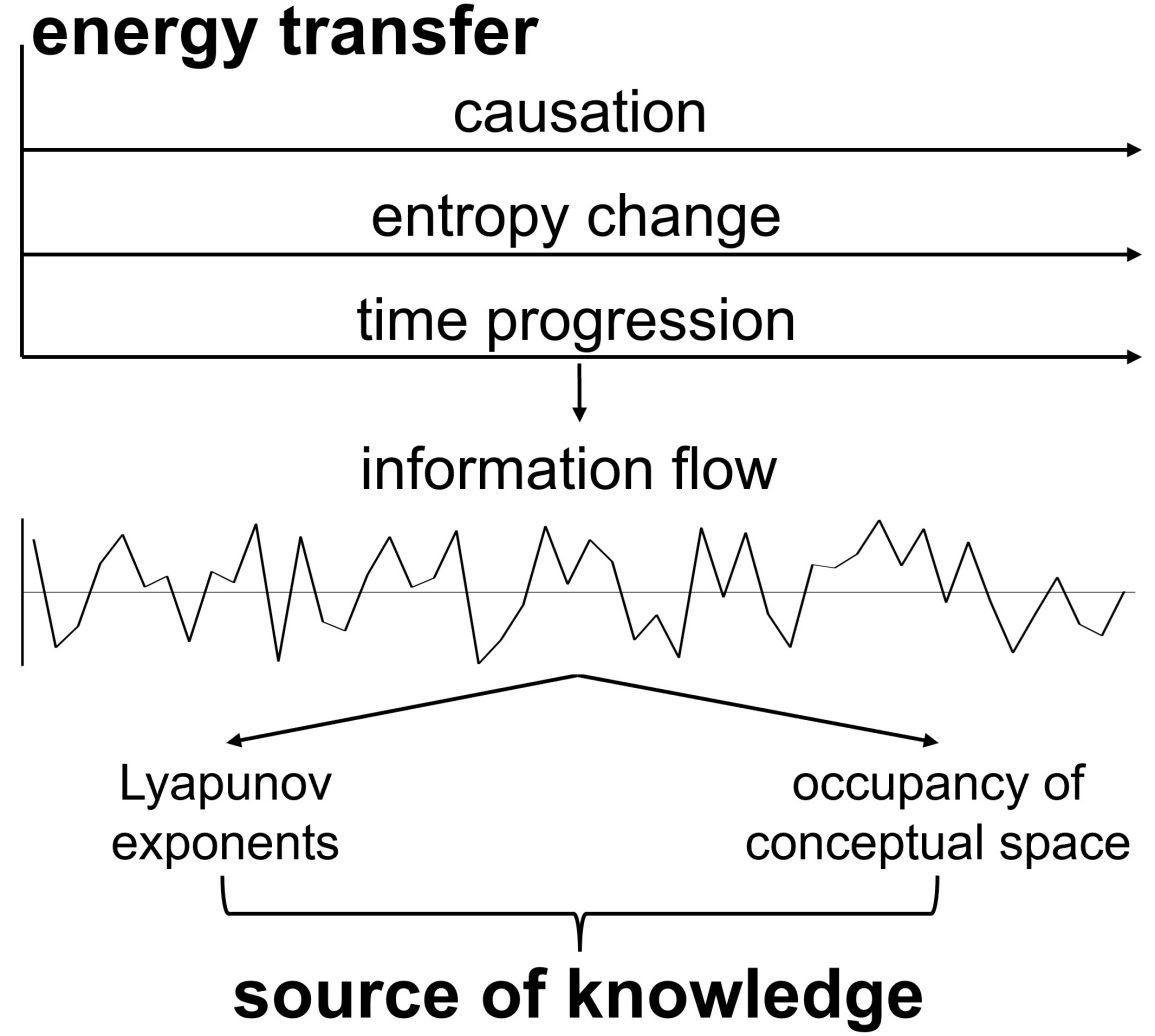
Causation, entropy, time progression, and information. Transfers of energy link causation, changes in entropy, and time progression to each other as well as to the evolution of attainable information: Both, causation and the progression of time, require the transfer of energy (also at the core of changes in thermodynamic entropy, which in emerging systems may decrease). The generation or loss of accessible information (data, evidence) about the occurrence, which is necessitated for knowledge, is quantifiable through Lyapunov exponents and information dimensions in conceptual space.

This investigation has elucidated connections by several physical entities to the evolution of attainable information, which is a cornerstone to the generation of knowledge. With every occurrence of a cause exerting an effect in the world, some amount of energy or momentum is transferred. This physical process also equates to an increment of time progression (the increase in thermodynamic entropy, prompted by the energy transfer, constitutes the advancement of an internal time). The information produced by this cause-effect occurrence via the removal of uncertainty—if captured as data or evidence through observation—can serve as a basis for scientific insight (Figure 1). However, all information has a limited lifespan: if not recorded by scientific inquiry, access to it will be lost due to dissipation (Roncaglia, 2019; Chiribella and Scandolo, 2015; Shaw, 1981). Given the dependence of knowledge on accessible information and the inevitable dynamics of information generation and obliteration in the macroscopic world, the habitually proclaimed “growth of knowledge” is an inaccurate veneration. Knowledge is fluid—it can be attained and bereaved.

The transfer of preserved properties (such as energy or momentum) is the driver of change in the world. It also produces an evolution (gain or loss) of accessible information. On the basis of the unitarity principle in quantum mechanics, information is also a preserved property; yet our ability to retrieve it is limited by the coarse-graining of our observations. Hence, the realization of knowledge is dependent on our access to information before it has dissipated. Various uncertainty principles (Weber, 2021) assure that such access is limited by laws of nature.

Scientific observation is tied to an observer. It is prompted, at its origin, by the formulation of a hypothesis. The ensuing collection of information (data, evidence) about an occurrence depends on the engaged methodology, and thus is a snapshot of this occurrence. Due to the probabilistic nature of information, the starting hypothesis of an inquiry is either weakened or strengthened by the preponderance of evidence. Hypotheses can never be proven to be correct [as envisioned in the concept of logical empiricism by members of the Vienna Circle (Hahn et al., 1929)] nor refuted by final proof [as envisioned by Karl Popper (Popper, 1982)]. An investigator-driven component in the processing of data, the interpretation through theories (mutually non-conflicting hypotheses on one subject), is always added in the procedure of inquiry. This is what turns information into knowledge. Statements devoid of information qualify as conviction or belief but not as knowledge. The present investigation does not venture beyond the realm of physics-based studies. The consumption of free energy in the least time may have bearing on the natural categorization of placing objects to classes (Lehmonen, 2022). It has been pointed out that a broader analysis of causation, beyond the natural sciences, may reveal deeper essential connotations (Xie, 2025, 2024).

The derivation from measurable information flows is key for the demarcation of knowledge from presumption. Yet, this dependence is also responsible for both uncertainty and incompleteness. Knowledge is incomplete, because dissipation on microscopic scales and the replacement of all old information by new information in emergent systems limit our access. Knowledge is uncertain, because our hypotheses and methodologies determine how we access information flows, and various uncertainty principles constrain the precision of our measurements.

Our natural perception that we exist in a space that comprises three axes (“dimensions”), height, depth and width, may have evolved more to aid our survival than to support our insights into the workings of the universe. Therefore, physical descriptions have frequently taken recourse to conceptual (abstract) spaces. As such, we utilize state space for modeling the possible configurations of a system, phase space for modeling the possible trajectories of an occurrence, and event space for vectoral depictions. Through their assignments of precise coordinates, conceptual state and phase spaces represent idealized depictions. Any actual event is bound to have a measurable and finite extent, both in time and in physical space. Its coordinates cover a range. Furthermore, the properties of time differ from the properties of space, most importantly because of the irreversibility of the former. While event space connects the execution of a process to the progression of time and to entropy generation (with time reversal being prevented by an infinite entropy barrier) (Prigogine, 1980), this space is non-continuous through the action of operators. For bringing together the equations of dynamics with thermodynamics, probabilistic (ensemble) methods are invoked to relate the average behavior of each particle to the overall behavior of the system. This averaging is successful because of the large number of particles involved in most processes. It now leads to the quantity of an internal time that represents the age of a dynamical system and reflects irreversible thermodynamical aspects. The adaptation of Lyapunov exponents from trajectories to vectors (Supplementary material 5) identifies an upper bound for the information dimension of the system.

Progress in multiple fields of research during the twentieth century has shaped our understanding of epistemology. Entropy—in thermodynamics as well as in information theory—has assumed a central role for explaining sources of knowledge. The laws of non-linear dynamics have built on insights of non-periodic flow with sensitive dependence on the starting conditions, emergence of properties from non-equilibrium states, and generation of new accessible information to fully replace old information. Through these developments, we have learnt to unify diverse branches of science at the expense of having to give up the absoluteness of scientific insight. The description of occurrences in conceptual space (depicting states, trajectories or vectors) has enabled a unification of deterministic and indeterministic descriptions of the world under the umbrella of complexity (Weber, 2013, 2019, 2020). Prominently, evidence supports the generation and obliteration of attainable information (Shaw, 1981). Now, the necessary interconnection among causation, time progression and entropy further refines our understanding of the sources for information and its processing into knowledge.
