# Supplementary material for: Sources of knowledge—causation begets entropy increase, time progression, and information flow
Source: Front Cognit. 2026 Apr 8;5:1795815. doi: 10.3389/fcogn.2026.1795815 (PMC13281034; doi:10.3389/fcogn.2026.1795815)
Supplement: Supplementary file 1 [file Supplementary_file_1.docx]

Sources of Knowledge –

Causation Begets Entropy Increase, Time Progression, and Information Flow

Supplementary Material

Supplement 1

Causation does not equate to determinism

The interpretation that occurrences have causes is derived from long-standing observations and interpretations. In 1949, Max Born sought to distinguish determination from causality (Born 1949). Determination meant that actual events are linked by laws of nature, such that certainly reliable predictions and retrodictions can be made from sufficient present data about them. He described two kinds of causation: nomic or generic causation and singular causation. Nomic causality means that cause and effect are linked by more or less certain or probabilistic general laws covering many possible or potential instances. An occasion of singular causation is a particular occurrence of a definite complex of events that are physically linked by antecedence and contiguity.

More recently, the model of a dichotomy between necessary and chance events (rolling a ball down a slope being an example for the former, and spinning a roulette wheel exemplifying the latter) has been widely accepted. Under this dichotomy, however, if there exists a formal link between determinism and indeterminism, neither of the two components can retain its character. This dualism in the description of the world, which inevitably creates two domains that are mutually exclusive opposites, clashes with our unitary experience (Weber 2021).

New questions have been raised in the study of complex non-linear systems, which are characterized by non-periodic flow with sensitive dependence on the starting conditions, emergence of properties from non-equilibrium states, and generation of new information to fully replace old information. Such occurrences, which are governed by non-linear differential equations, have traditionally been considered as deterministic on the basis of their driving algorithms, even though the slightest, immeasurable deviations in their initial states can lead to dramatically different outcomes (Lorenz 1963). In the progression of complex occurrences there arise decision points, at which infinitesimal influences can propel events onto one of two (or more) very different future paths. For such systems, uncertainty about the future grows rapidly, because there are fundamental limits to how precisely we can describe their evolution. For these equations, non-periodic solutions cannot readily be determined, except by numerical procedures. Proceeding in this manner, the emergence of ordered structures from non-equilibrium conditions has been described in physics, chemistry, and evolution. Their foundation in chance or determinism has been subject to debate.

There are algorithms to aid in the breakdown of the distinction between necessity and chance. Objects and occurrences have varying degrees of complexity (previously perceived as variable abundance of chance elements). At the bifurcations (decision points, tipping points) in complex events (the edge of a flipped coin being one example), it is principally impossible to measure with sufficient precision whether the choice between one or the other future path is governed by deterministic or indeterministic mechanisms.

- Non-linear systems research often describes events as trajectories in phase space. Such phase space is divisible into blocks of minimum size that represent states. Their expansions constitute the limits to the precision of obtainable knowledge. Efforts to more accurately measure the system by reducing the block size eventually lead to the microscopic scales, wherein the Heisenberg uncertainty principle assures that the more precisely the position in phase space is determined the less precisely the velocity can be assessed. The analogous relationship exists between energy and time. Therefore, phase space models have minimally discernible resolution, description beyond which is infeasible (Shaw 1981).

- Thermodynamic considerations striving to incorporate irreversibility into physical law have developed event space models, wherein the resolution of the description is restrained by the eigenvectors and eigenvalues of the operators (Prigogine 1980). Again, there is insufficient resolution at the bifurcation points for a dichotomous assessment of chance or necessity.

What has, in older conceptual frameworks, been described as the abundance of chance elements (the flip of a coin is less random than the throw of a die, which is less random than the spin of a roulette wheel) actually are manifestations in degrees of complexity. This complexity is mathematically tractable (Weber 2021). Notwithstanding the connections between causes and effects, the world is governed neither by deterministic nor by indeterministic principles and there is no dichotomy between those two.

- While cause leads to effect, this effect is not equal to a determined and precisely predictable outcome.

- What colloquially is described as chance elements are actually degrees of complexity (Weber 2019, Weber 2020).

Supplement 2

Diverse manifestations of entropy are interconnected

In 1798, Benjamin Thompson discovered that work could be transformed into heat without limit (this would lay the foundation for the first law of thermodynamics, which postulates the conservation of energy). Based on groundwork by Sadi Carnot in the 1820s, the concept of entropy (a continually decreasing amount of available free energy) was introduced in 1865 by Rudolf Clausius. He described it as the transformation content (dissipative use of energy) of a thermodynamic system or working body of chemical species during a change of state.

The information content of a process is interpretable as the removal of the extent of uncertainty that exists before its execution. The information content of an event is principally quantifiable in the algorithms developed by Claude Shannon (Shannon 1948). An event with a certain outcome (a probability of 1) yields no new information. With its execution, the random element is removed, and new information is communicated, which is quantifiable as information entropy.

The formal convergence between the Shannon formula for entropy in information theory and the Boltzmann formula for entropy in thermodynamics has given rise to numerous debates over meaningful connections between them (Hejna and Šesták 2024). While multiple interpretations of and formulas for entropy have been developed, it has become clear that thermodynamic entropy is connected to information entropy.

- They need to satisfy common requirements and have a common origin in probability theory. “If one accepts the probabilistic interpretation of the entropy, and agrees on the meaning of Shannon’s information, then the interpretation of the thermodynamic entropy as thermodynamic information becomes inevitable” (Ben-Naim 2008). Being interpretable as thermodynamic information, entropy expresses the amount of information needed to specify the state of a system to a particular level of accuracy.
- The macroscopic state of a system is characterized by a distribution on the microstates. The Gibbs entropy (holding for all systems) is a generalization of the Boltzmann entropy (developed for systems in global thermodynamic equilibrium) (Gao, Gallicchio et al. 2019). While both are quantifications of the microstates available to a system, the Gibbs entropy does not require the system to be in a single well-defined macrostate. This formula is the basis for the information-theoretical interpretation of thermodynamics and thus forms a bridge to the Shannon entropy [Equation S1].

$S= -k_{B}\sum_{i} p_{i}ln(p_{i})$ (S1)

where k_B_ is the Boltzmann constant, while the sum is over all possible microstates i, with p_i_ the corresponding probability of the specific microstate.

- In one viable interpretation, entropy reflects the expression for the amount of information needed to specify the state of a system to a particular accuracy. Landauer’s principle (Landauer 1961, Plenio and Vitelli 2001) pertains to the theoretical lower limit of energy consumption by the process of computation (at 20^o^C, approximately 2.805 x 10^-21^ J). It stipulates that any logically irreversible processing of information must be accompanied by a corresponding entropy increase in non-information-bearing degrees of freedom by the information-processing apparatus or its environment [Equation S2].

$E \geq k_{B} T\ln2$ (S2)

where E = energy required for the computation, k_B_ = Boltzman constant, T = temperature (Kelvin). In generalized terms, any information that has a physical representation must somehow be embedded in the statistical mechanical degrees of freedom of its physical system. This principle combines thermodynamic and information theoretic applications of entropy.

Other formulations of entropy have been put forward, which measure complexity in dynamical systems (Kolmogorov-Sinai entropy) or in strings of numbers (Kolmogorov-Chaitin complexity). They share with the Shannon entropy the dependence on information. Whereas Shannon entropy is focused on the missing information, Kolmogorov-Chaitin complexity is focused on the content of information.

**Information entropy has syntactic and semantic components**

For the attainment of knowledge, not all removal of uncertainty is relevant, but only the fraction of it that is pertinent to the question under study. The traditional assessment of information does not discriminate meaningful information from all other information. In the General Theory of Information, the ontological principles make a distinction by separating information in general from information for a system *R*. Empirically, it is possible to speak only about information for a system. Information for a system *R* is a capacity to cause changes in the system *R* (Burgin 2017). In the semantic information theory of Bar-Hillel and Carnap, information causes change in knowledge about the real state of a system under consideration (Bar-Hillel 1958). Meaningful information is now referred to as syntactic information, which reflects the amount of statistical correlation between systems. By contrast, semantic information delineates those correlations, which carry significance or meaning for a given system. Semantic information is the subset of syntactic information, which a physical system has obtained about its environment, and which is necessary for this system to maintain its own existence (equating to the maintenance of a low entropy state). Non-equilibrium statistical physics can express semantic information in thermodynamic terms (Maroney 2009). It is possible to define a viability function (a real-valued function that quantifies the system’s existence at a given time) as the negative Shannon entropy of the distribution over the states by the system (Kolchinsky and Wolpert 2018).

**von Neumann entropy is uniquely distinct but connected**

Quantum entropy may be the most fundamental manifestation. An inference from von Neumann entropy has implications for how the large-scale world emerges from the quantum world (entanglement is the first step in the transition from the quantum to the classical world – the process of decoherence). The evolution of entanglement connections may drive the second law of thermodynamics. However, some specifics are unique.

Whereas thermodynamic entropy is a state function on the space of macrostates, not on microstates or wavefunctions, von Neumann entropy in quantum mechanics is defined on the quantum state itself [Equation S3]. Represented by a density operator $\rho$, it is given by

$S_{\mathrm{vN}}(\rho)=-k\text{ }\mathrm{Tr}(\rho\ln\rho)$ (S3)

Quantum entropy is therefore a functional on the state space of quantum mechanics, not on thermodynamic state variables.

In quantum mechanics, probabilities are intrinsic to the formalism. Mixed states may arise from ensembles or from entanglement with an environment, even when the global state is pure. Thus, von Neumann entropy can increase without any loss of information globally, simply by tracing out degrees of freedom. A key conceptual difference from thermodynamic entropy is that quantum entropy can be local while information is globally conserved.

Regarding time evolution and irreversibility, for an isolated quantum system evolving unitarily, it holds that

$\frac{d}{dt}S_{\mathrm{vN}}(\rho)=0$ (S4)

von Neumann entropy is time‑invariant under Schrödinger evolution [Equation S4]. Irreversibility appears only when subsystems are considered or measurements, decoherence, or coarse‑graining are introduced. An elemental change boils down to a quantum, such as a photon, carrying energy on its period, moving from a system to its surroundings, or vice versa, thereby consuming free energy.

For large, thermalized quantum systems described by Gibbs [Equation S5] states

$\rho=\frac{e^{-\beta H}}{Z}$ (S5)

the von Neumann entropy numerically equals the thermodynamic entropy. In this sense, von Neumann entropy can be called a quantum generalization of thermodynamic entropy. Yet, both concepts still differ, because

- thermodynamic entropy refers to macroscopic irreversibility
- von Neumann entropy refers to state uncertainty or entanglement
- the former generates time asymmetry; the latter preserves time symmetry under unitary dynamics.

Thermodynamic entropy measures irreversible macroscopic evolution toward equilibrium, while von Neumann entropy measures the mixedness or entanglement of a quantum state and is conserved under unitary dynamics; they coincide only for large, thermalized systems, not in general.

Supplement 3

Past, present and future are distinct

Various concepts of time advancement have been introduced by classical mechanics, physics of the microscale, relativity theory, and thermodynamics. In Newton’s physics, the present is no more than the connection between the arrow from the past and the arrow to the future. It has no extension. Modern physics, including quantum theory and relativity theory, has provided only limited models of temporal development. The past and the future are described as trajectories in phase space, which implies that both are somehow contained in the present. Quantum mechanics has introduced a probabilistic description on the microscopic level, striving to shield explanations from random fluctuation. The macroscopic thermodynamic description typically focuses on mean values, for which random fluctuations become negligible.

We perceive the present as having an extension (this moment, today, the current year, etc.). Although experienced as real in our daily lives, mechanics defines the present only in a narrow surrounding of where it is being described. “The notion of ‘now’ is nothing more than a certain relation between a certain observer and the rest of the universe” (Gödel 1949). Attempts have been made to account for extensions of time periods.

- An extent of duration was introduced by the recognition that time may not progress smoothly as a continuum. Planck time (~ 5.4 × 10^−44^ seconds) is the unit of time in the system of natural units. Established physical theories are believed to fail at this time scale, and a prevailing hypothesis in physics expects that the Planck time might be the smallest unit of time that could ever be measured, even in principle. Tentative physical theories that describe this time scale exist in loop quantum gravity and others. Planck time represents the minimum extension of ‘now’.

- In relativity, the temporal order of the universe is partial, not complete (Einstein 1916). Every event is associated with light cones; light travels along the oblique lines that define its reach. The structure of the light cones can be such that a trajectory advancing always toward the future can return to the point in space-time where it began, thus generating closed temporal lines (Lewis 1976). The expanded present is the set of events that are neither past nor future (in relation to every event, there is its past, its future, and part of the universe that is neither past nor future).

However, without the incorporation of thermodynamic considerations, the accounting for a tangible present has been incompletely successful.

In order to suitably represent our experience of time progression in the world, there has been a need to expand the formulas of dynamics/quantum mechanics to include the asymmetrical events of thermodynamics, so as to reflect the distinction among past, present and future. Our clocks measure time progression under conventional standards, which defines an external time (Prigogine 1980). The progression of this canonical time, the label of dynamics, can be linked to thermodynamics as an average over the internal operator times that equate to increases in entropy. In this framework, entropy change is causative for an internal time, which is distinct from the time concept that in classical or quantum mechanics simply labels trajectories or wave functions. The present has acquired a tangible expansion, defined by the eigenvectors and eigenvalues of the time operator. The concept of time has changed with the insights of complexity research from parameter time via coordinate time to operator time (Prigogine 1975).

Irreversible occurrences are the essence of time progression

Through aging and the limitation of existence by death, all living creatures experience the irreversibility of time. Yet, any operational definition of time does not address why occurrences can happen forward and backward in space, whereas they only happen in the forward progression of time. Therefore, more profound definitions of time are required to account for the experience of irreversibility, the experience that time progresses in one direction.

- Traditional mechanics studied processes as reversible and coined the term “time symmetry” (“time reversal symmetry”) to describe this assumption. Symmetries (including time reversal symmetry) have been central to classical physics: The laws of motion – Newtonian as well as quantum mechanics – do not distinguish between past and future.

- Relativity theory showed that the progression of time is not uniform but is influenced by motion. Still, the impossibility of time reversal was insufficiently represented, and entropy was not accounted for.

- Contrastingly, spontaneous symmetry breaking, which is a fundamental non-linear process, plays an important role in many areas of modern physics. Symmetry breaking gives mass to elementary particles in the unified theory of electromagnetic and weak interactions, it also plays a pivotal role in the inflation theory regarding the expansion of the early universe.

- To close existing gaps, a unification of dynamics and thermodynamics has described time as an operator and has tied it to entropy. The energy transfer that is at the core of causation effectuates the progression of an operator time, such that time reversal is prevented by an infinite entropy barrier (Prigogine 1980). This internal time now embodies the mathematical characteristics of an operator and has lost continuity as well as reversibility (Prigogine 1975, Gialampoukidis and Antoniou 2015).

Supplement 4

Entropy may be linked to canonical time

Attempts have been made to link canonical time to entropy through Boltzmann’s H-theorem and via an operator time that progresses internally within physical processes. In a distinct application, an arrow of time has been derived from transfer entropy.

Related to, but separate from entropy, Boltzmann’s H-theorem [Equations S6-S8] defines a function H(t) that decreases with time and assumes a minimum once equilibrium is reached.

$H\left( t \right)= \int f\left( v,t \right)\log\left[ f\left( v,t \right) \right]dv$ (S6)

Basically, it holds that,

$\frac{dH(t)}{dt}\leq0$ (S7)

and at equilibrium with t →∞,

$\frac{dH(t)}{dt}= 0$ (S8)

Boltzmann supposed that the behavior of the function -H(t) would be the same as that of entropy, which meant that the entropy always increased with time, and at equilibrium reached a maximum.

Systems far from steady state may become unstable when they are driven further toward non-equilibrium. They reach a crisis (a bifurcation point), beyond which new and highly organized states can appear. Self-organization may prod the spontaneous development of structures. Such dynamical states are not associated with minimal entropy production by the system, but the entropy produced is exported to the external environment. Hence, on a local level, the concept of an arrow of time, which equates to uniform degeneration into thermodynamic equilibrium, is in need of adjustment. Suitable mathematical models are based on equations that show how various observable properties of a system change, thus accounting for a unique concept of time. Entropy and internal time assume the propensity of operators [Equation S9]

$i\frac{\partial\tilde{\rho}}{\partial t}= \Lambda^{-1}L\Lambda\tilde{\rho}$ (S9)

$\tilde{\rho}$= state of the system, L = Liouville operator, $Ʌ^{-1}$ = time operator (also written as T). The recognition that the direction of time is caused by an infinite entropy barrier against time reversal has unified the symmetrical and asymmetrical aspects of nature (Prigogine 1975, Prigogine 1980). The operator time T is closely related to the microscopic entropy operator M. T has as eigenvalues the possible ages a system may have. Thus, a novel meaning for time emerges that is associated with evolution. In general, a distribution has no well-defined age but may be expanded in a series of functions having well-defined ages. M is defined as a microscopic entropy operator that does not commute with the Liouville operator L (Prigogine 1975).

More recently, an arrow of time has been derived from the analysis of transfer entropy and its associated evolution of information. Transfer entropy (Schreiber 2000, Paluš, Komárek et al. 2001) is a non-parametric statistic in information theory, which quantifies the directional transfer of information between two processes. The algorithm detects asymmetrical information flow by measuring how much the knowledge of one system’s past improves the prediction of another system’s future beyond what could be predicted using only the second system’s past. For systems that process information, such as those undergoing feedback, a robust arrow of time can be formulated by considering both the apparent physical behavior, which leads to conventional entropy production, and the information dynamics, which leads to an information-theoretic arrow of time (Spinney 2016).

Supplement 5

Lyapunov exponents are applicable to vector spaces

# The redefinition of entropy and an internal time in the form of operators (Prigogine 1975, Prigogine 1980) has been a substantial change. Due to the reliance on the eigenvectors and eigenvalues of the operators for time and thermodynamic entropy, an assessment of the information evolution associated with an event now requires algorithms that are independent of trajectories in phase space. To achieve this, a reformulation of the Lyapunov characteristic exponent has been applied to the vector-based event space. The application of the multiplicative ergodic theorem to this event space is feasible with the use of vector norms. It yields a readout for the information evolution of an event and lays the foundation for a calculation of dimensionality that is directly inferred from the Lyapunov characteristic exponents associated with the process under study (Weber 2019, Weber 2020). The insertion of the Lyapunov characteristic exponent, calculated for event space (a vector space), into the formula for the Lyapunov dimension achieves the connection of a process, the execution of which impacts internal time and thermodynamic entropy, to the fractal dimension of the event space occupied by the occurrence, and to its information entropy.

**References**

Bar-Hillel, Y., Carnap, R. (1958). "Semantic Information." The British Journal for the Philosophy of SciencePhysical Review E **4**: 147-157.

Ben-Naim, A. (2008). A farewell to entropy: Statistical thermodynamics based on information. Singapore, World Scientific Publishing Corporation

Born, M. (1949). Natfral Philosophy of Cause and Chance. Oxford, The Clarendon Press.

Burgin, M. (2017). "The general theory of information as a unifying factor for information studies. The noble eight-fold path." Proceedings **1**: 164.

Einstein, A. (1916). "Die Grundlage der allgemeinen Relativitätstheorie." Annalen der Physik **49**: 769-822.

Gao, X., E. Gallicchio and A. E. Roitberg (2019). "The generalized Boltzmann distribution is the only distribution in which the Gibbs-Shannon entropy equals the thermodynamic entropy." The Journal of Chemical Physics **151**: 034113.

Gialampoukidis, I. and I. Antoniou (2015). "Entropy, age and time operator." Entropy **17**: 407-424.

Gödel, K. (1949). "An example of a new type of cosmological solutions to Einstein’s field equations of gravitation." Reviews of Modern Physics **21**: 447-450.

Hejna, B. and J. Šesták (2024). "Interrelatedness of thermodynamics and information: transformation of heat as a measurable information process and quantity, an overview." Journal of Thermal Analysis and Calorimetry **149**: 11517–11528.

Kolchinsky, A. and D. H. Wolpert (2018). "Semantic information, autonomous agency and non-equilibrium statistical physics." Interface Focus **8**: 20180041.

Landauer, R. (1961). "Irreversibility and heat generation in the computing process." IBM Journal of Research and Development **5**: 183–191.

Lewis, D. (1976). "The paradoxes of time travel." American Philosophical Quarterly **13**: 145-152.

Lorenz, E. N. (1963). "Deterministic nonperiodic flow." Journal of the Atmospheric Sciences **20**: 130-141.

Maroney, O. J. E. (2009). "Generalizing Landauer’s principle." Physical Review E **79**: 031105.

Paluš, M., V. Komárek, Z. Hrnčíř and K. Štěrbová (2001). "Synchronization as adjustment of information rates: Detection from bivariate time series." Physical Reviews E **63**: 046211.

Plenio, M. B. and V. Vitelli (2001). "The physics of forgetting: Landauer’s erasure principle and information theory." Contemporary Physics **42**: 25-60.

Prigogine, I. (1975). "Dissipative structures, dynamics, and entropy." International Journal of Quantum Chemistry **9**: 443-456.

Prigogine, I. (1980). From Being to Becoming: Time and Complexity in the Physical Sciences. New York, NY, USA, W.H. Freeman and Company.

Schreiber, T. (2000). "Measuring information transfer." Physical Review Letters **85**: 461-464.

Shannon, C. E. (1948). "A mathematical theory of communication." Bell Systems Technology Journal **27**: 379–423, 623–656.

Shaw, R. (1981). "Strange attractors, chaotic behavior, and information flow." Zeitschrift fuer Naturforschung **36**: 80-112.

Spinney, R. E. L., J. T.; Prokopenko, M. (2016). "Transfer entropy in physical systems and the arrow of time." Physical Review E **94**: 022135.

Weber, G. F. (2019). "Information gain in event space reflects chance and necessity components of an event." Information **10**: 358.

Weber, G. F. (2020). "Information dynamics in complex systems negates a dichotomy between chance and necessity." Information **11**: 245.

Weber, G. F. (2021). How Complexity Shapes the World. Cambridge, Cambridge Scholars Publishing.
